# Exploring Vaccine Hesitancy in the Philippines: A Content Analysis of Comments on National TV Channel YouTube Videos

**DOI:** 10.3390/ijerph22060819

**Published:** 2025-05-22

**Authors:** Daniel Fritz Silvallana, Carlos Elias, Daniel Catalan-Matamoros

**Affiliations:** 1 Department of Communication and Media Studies, Davao del Norte State College, Panabo City 8105, Philippines; danielfritz.silvallana@dnsc.edu.ph; 2 School of Communication and Creative Arts, Deakin University, Burwood, VIC 3125, Australia; 3 Department of Communication and Media Studies, Madrid University Carlos III, 28903 Madrid, Spain; dacatala@hum.uc3m.es

**Keywords:** vaccine hesitancy, COVID-19, YouTube, Philippines

## Abstract

Examining public attitudes towards COVID-19 vaccination is crucial for understanding the global effort to combat the ongoing pandemic. Social media platforms such as YouTube play a significant role in the dissemination of information and misinformation about the vaccine, making it imperative to analyze user comments to gain insights into vaccine perceptions. Analyzing the Philippines case is particularly significant as it provides insights into the attitudes towards COVID-19 vaccination in a country that has been heavily impacted by the pandemic. The current study investigates the discourse surrounding vaccine hesitancy in comments on YouTube videos announcing the COVID-19 vaccination campaign by the Philippines national TV channels and its impact on engagement levels. A total of 741 YouTube comments were analyzed, with 80% exhibiting vaccine-hesitant related discourse. The results indicate that those with negative attitudes towards COVID-19 vaccination exhibit higher engagement levels than those supporting vaccination (*p* < 0.05). Additionally, the most commonly used themes in vaccine-hesitant posts were “ingredients”, “health department control”, “pharmaceutical interest”, and “adverse effects”. Moreover, 134 sources were identified among the posts, with vaccine-hesitant posts utilizing more sources than supportive vaccine posts (*p* < 0.001). The most significant information sources utilized in the posts were related to other YouTube users, politicians, clinicians, and scientific papers. Finally, a total of 890 discourses were coded, with the most frequently used discourse types among vaccine-hesitant posts being negationist, institutional, preventive, political, and pharmaceutical. These findings offer valuable insights into the nature and prevalence of vaccine hesitancy discourse on social media platforms and its impact on public engagement. This study highlights the importance of targeted communication strategies and the provision of accurate information from reliable sources in addressing vaccine hesitancy.

## 1. Introduction

Vaccination is an effective measure to combat infectious diseases, and it has been recognized as one of the most successful public health interventions in history [1]. With the outbreak of COVID-19, the development of vaccines has become a global priority, and vaccination has been hailed as a crucial tool to end the pandemic. However, the success of vaccination programs is heavily dependent on the public’s willingness to get vaccinated. Vaccine hesitancy, defined as the reluctance or refusal to vaccinate despite the availability of vaccines, has become a significant barrier to achieving herd immunity against COVID-19 [2].

Social media platforms such as YouTube play a significant role in shaping public attitudes towards vaccination [3,4,5]. They provide a forum for individuals to share their opinions and experiences regarding vaccination, making them a valuable source of data for researchers seeking to understand vaccine perceptions [6]. Analyzing user-generated content on YouTube can provide insights into the reasons behind vaccine hesitancy and how it is expressed by the public.

Previous research has examined the role of social media platforms in the dissemination of information about COVID-19 and its associated vaccines. Specifically, studies have focused on analyzing user-generated content on YouTube related to COVID-19 vaccination. For example, a study by Uddine and Islam [7] analyzed 500 YouTube videos related to COVID-19 vaccines and found that the majority of the comments were positive towards vaccination. However, the authors noted that misinformation and conspiracy theories were present in a small proportion of the comments, highlighting the potential impact of such content on vaccine uptake. Analyzing attitudes towards COVID-19 vaccination is critical for understanding the global efforts to combat the pandemic. Given the widespread dissemination of information and misinformation through social media platforms like YouTube, it is particularly important to examine comments made by users to gain insight into public perceptions of the vaccine. The current study builds upon this previous research by analyzing the discourse surrounding vaccine hesitancy specifically in the context of the Philippines and its impact on engagement levels.

The Philippines is one of the countries heavily impacted by the COVID-19 pandemic. As of 13 April 2024, the country has recorded over 4.1 million cases (3.53% of the national population) and over 60,000 deaths (0.052% of the national population) [8]. Some factors that may have contributed to the high number of COVID-19 cases and deaths in the Philippines include a dense population, limited healthcare infrastructure, and challenges in implementing effective public health measures such as contact tracing and testing. Additionally, the country’s high poverty rate and the prevalence of informal settlements may have made it difficult for people to adhere to quarantine and social distancing measures, contributing to the spread of the virus. Finally, the emergence of new COVID-19 variants and the slow rollout of the vaccine in the country may have also contributed to the ongoing impact of the pandemic. The Philippine government has implemented various measures to curb the spread of the virus, including a vaccination campaign that aimed to vaccinate 70% of the population by the end of 2022 [9]. However, vaccine hesitancy has been a significant challenge in the Philippines. A study conducted by the World Health Organization [2] reported that only 32% of Filipinos were willing to receive the COVID-19 vaccine. Even with the government’s efforts to encourage public participation in its COVID-19 vaccination initiative, it has been clearly observed that vaccine hesitancy persists as a significant obstacle in the Philippines [10,11].

YouTube is one of the most popular social media platforms in the Philippines, with a reported 57.7 million active users as of 2025 [12]. It is used for various purposes, including entertainment, education, and information sharing [13]. YouTube is becoming an increasingly important platform for Filipinos to access information about health and healthcare. In a study conducted by Jimenez-Santos et al. [14], YouTube was found to be a commonly used platform for Filipinos seeking health information. The study found that the most searched topics were health conditions and diseases, followed by medication and treatment information. Additionally, social media platforms such as YouTube have been shown to be an important source of information for Filipinos during public health emergencies, such as the COVID-19 pandemic [15]. This highlights the potential of YouTube to influence public perceptions of health-related issues and underscores the importance of analyzing user-generated content on the platform to gain insights into vaccine hesitancy in the Philippines. In addition, studying vaccine hesitancy in comments posted on YouTube has been found to be a good strategy taking into account the large number of anti-vaccine users that interact on YouTube [16,17]. Indeed, despite the efforts by Facebook and YouTube, COVID-19 vaccine-related misinformation in the form of anti-vaccine videos propagated on both platforms during the national vaccination campaigns [5].

Given the importance of understanding vaccine hesitancy in the Philippines, this study aims to investigate the discourse surrounding vaccine hesitancy in comments on YouTube videos announcing the COVID-19 vaccination campaign in the Philippines as a case, thereby providing insights for future work on vaccination campaigns. This study focuses on YouTube comments because they are a valuable source of citizen-generated content and have been used in previous studies to investigate vaccine hesitancy [18,19]. This study will try to answer the following specific research questions:

- RQ1: Do vaccine-hesitant posts receive a higher engagement level?
- RQ2: What themes are more frequently used in vaccine-hesitant posts?
- RQ3: Do vaccine-hesitant posts use more sources than vaccine-supportive posts? What sources are most frequently used by vaccine-hesitant posts?
- RQ4: Are there differences in the public discourse of vaccine-hesitant posts and those promoting vaccination? What are the most frequent discourses used by vaccine-hesitant posts?
- RQ5: What are the most frequently referred mentions in the posts?

## 2. Methodology

This study employs a content analysis design to examine the comments obtained from YouTube videos published by national TV channels in the Philippines that announced the nationwide COVID-19 vaccination campaign. This study focused on identifying the news videos where government officials presented the COVID-19 immunization campaign in the Philippines. Both GMA and CNN national channels were searched. These channels were selected for two reasons. First, both TV channels have wide market reach and viewership. The GMA Network is one the Philippines’ largest media conglomerates, while CNN Philippines, though having a smaller overall audience, captures a significant portion of the urban, educated demographic. Hence, these networks represent news consumption patterns across different socio-economic segments of the Philippine society. Second, these channels have easy digital accessibility as they both maintain YouTube channels that preserve their broadcasts. Their YouTube channels serve as publicly accessible records, creating a permanent repository of public discourse that can be methodically analyzed.

The videos analyzed in this article were originally published on the YouTube channels of CNN Philippines on 28 February 2021, and GMA News on 1 March 2021. The CNN Philippines video features coverage of the government’s official presentation of the COVID-19 immunization campaign in the Philippines. The video also includes discussions on President Duterte’s call for the public to get vaccinated, highlighting the reassurances of Filipino experts regarding the efficacy of the initial batch of COVID-19 vaccines arriving from a Chinese manufacturer. Meanwhile, the GMA News video showcases the government’s announcement of the COVID-19 vaccination campaign in the Philippines. The video is part of the nightly news program called “24 Oras”. It offers comprehensive coverage of the arrival of Sinovac vaccines in the Philippines, featuring interviews with government officials and health experts. The collection of comments took place on 22 November 2021.

The CNN video remains accessible following CNN Philippines’ closure on 31 January 2024 via NewsWatch Plus, while the GMA News video has since been removed or made private. The following are the URLs with access dates of the two media channels and videos analyzed in this study:

- CNN Philippines video: titled “Vaccination starts today with arrival of first vaccines” [https://www.youtube.com/watch?v=aDSXIANZ9KY], last accessed date (20 April 2025).
- GMA News video: titled “24 Oras” [https://www.youtube.com/watch?v=_qADjU3JpRQ&t=0s], last accessed date (25 November 2021).

Furthermore, this study analyzed engagement metrics including likes and replies from videos. Social media engagement refers here to “user’s interactions, reactions, and connections with content”, highlighting volume as a proxy for visibility and discourse participation [20]. Hence, we measured engagement metric as quantitative activity and not sentiment, although we conducted sentiment analysis separately during qualitative coding. Initially, a total of 873 comments were collected, with 729 gathered from GMA News and 144 from CNN Philippines. After excluding 132 comments that did not meet the inclusion criteria (comments unrelated to the video theme or the vaccine discourse, such as those containing commercial content or unrelated emoticons), a final set of 741 comments was analyzed.

All of the comments in each sample were independently coded by one researcher (DFS) following initial training sessions covering codebook interpretation, practice coding on sample materials, and discussion of discrepancies and clarification of coding rules. While a complete double-coding of all comments was not conducted due to resource constraints, we randomly selected 10% of comments across two video samples coded by two researchers. The inter-rater reliability using the Kappa coefficient test was found to be high (κ = 0.969). Metadata were gathered for each video, including date uploaded, source, length (in minutes), and number of views. A form to code each of the comments was developed for this study and included the variables as seen in Table 1.

The analysis comprised frequency and percentage distributions for dichotomous content variables. To assess the engagement level as a continuous variable, we adopted a method that involved aggregating the number of likes and replies received in each comment. Analysis was conducted within each of the successive samples using SPSS software (version 25.0; IBM Corp., Madrid, Spain). To investigate significant differences between variables, we employed the Mann–Whitney U test, a non-parametric statistical test suitable for analyzing ordinal or continuous variables. This test allowed us to assess the presence of statistically significant variations between the variables under examination, providing key insights into the relationships and associations within the dataset.

In relation to ethical aspects, analyzing publicly available YouTube comments in this research study does not require an ethical committee review for several reasons. Firstly, the comments are already accessible to the public and can be freely viewed and examined by anyone without any restrictions. Therefore, there is no invasion of privacy or confidentiality concerns since the individuals who posted the comments have willingly made them public. Secondly, the research organization where this study was conducted has specific guidelines in place that exempt studies from ethical committee review if they do not involve human subjects. Since the YouTube comments are considered public text data, they fall outside the scope of ethical review.

## 3. Results

The total sample was composed of 741 comments, of which 593 (80%) showed vaccine-hesitant-related discourse. From these, 46 (8%) fully refused the vaccine, while 131 (22%) showed concerns and doubts about it, and 416 (70%) showed other types of hesitant behaviors (delayed vaccination, selective vaccination, vaccine complacency, etc.).

In order to answer RQ1, a Mann–Whitney U test was performed to evaluate whether engagement level differed by vaccine-hesitant discourse in the post. The results indicated that there were significant differences, with comments expressing vaccine hesitancy soliciting significantly more engagement than those supporting vaccination (*p* < 0.05).

In order to answer RQ2, to identify the most frequently used themes in vaccine-hesitant posts, we coded 730 themes in those posts that showed vaccine hesitancy. Please note that, in some comments, we coded two different themes. The most frequent themes were “ingredients of vaccines” (25%), “health department control” (21%), “pharmaceutical interest” (20%) and “adverse effects” (18%). See Table 2.

In relation to RQ3, 134 sources were identified among the posts. There are significant differences (*p* < 0.001) in relation to the use of sources, as vaccine-hesitant posts used largely more sources than supportive vaccine posts. In fact, only two sources related to the “websites” category were used by non-hesitant comments Table 3. In addition, Table 4 shows that the most important sources of information used in the posts are related to other Youtube users (31%), politicians (19%), clinicians (10%), and scientific papers (8%).

In total, 890 discourses were coded. In relation to vaccine hesitancy, the most frequently used discourse types were negationist (16%), institutional (14%), preventive (10%), political (9%), pharmaceutical (7%), and questioning (7%) Table 5.

Lastly, the posts were systematically coded to capture any mentions of other users, addressing RQ5. Table 6 presents the findings, highlighting that mentions of government representatives were the most frequently used.

## 4. Discussion

Our study focuses on examining the discourse surrounding vaccine hesitancy within comments posted on YouTube videos announcing the COVID-19 vaccination campaign by national TV channels in the Philippines. Several key findings have emerged from our analysis. Firstly, vaccine-hesitant comments received higher levels of engagement compared to those expressing support for vaccination. Secondly, we identified recurring themes within the comments, including discussions on the “ingredients of vaccines”, “health department control”, “pharmaceutic interests”, and “adverse effects”. Thirdly, we observed that vaccine-hesitant comments relied on a wider range of informational sources, with “YouTube users”, “politicians”, “clinicians”, and “scientific papers” being the most frequently cited sources. Fourthly, among vaccine-hesitant comments, we identified distinct discourse types, including negationist, institutional, preventive, political, pharmaceutical, and questioning. Finally, our analysis revealed that mentions of government representatives were the most commonly found within the comments.

Our study showed how vaccine-hesitant comments received significantly higher levels of engagement compared to those expressing support for vaccination. This finding is consistent with previous research suggesting that vaccine-related discussions often generate polarized opinions and intense interactions [21,22,23]. One plausible explanation for the observed hesitancy and skepticism in our findings could be traced to the Dengvaxia controversy in the Philippines, which created deep-seated mistrust toward vaccination programs, health institutions, and pharmaceutical companies [24]. In 2017, the Philippine government implemented a school-based dengue vaccination program using Dengvaxia, the world’s first licensed dengue vaccine. However, the vaccine manufacturer Sanofi Pasteur announced later that the vaccine could increase the risk, which led to several investigations of deaths allegedly linked to the vaccine. The controversy resulted in widespread “social trauma” among Filipinos, creating profound mistrust and fear toward both state authorities and health institutions [10,11]. These results also align with the concept of “negativity bias”, wherein controversial or dissenting viewpoints tend to attract more attention and elicit stronger reactions [25]. These findings have important implications for public health communication strategies, emphasizing the need for targeted interventions to counteract the disproportionate influence of vaccine-hesitant perspectives in online platforms.

Our analysis identified several recurring themes within the comments received, shedding light on the concerns and topics that dominated vaccine-related discussions. These themes encompassed discussions on the “ingredients of vaccines”, “health department control”, “pharmaceutic interests”, and “adverse effects”. These findings mirror trends observed during the SARS-CoV-2 pandemic, where global vaccine acceptance rates reached 75.2% in mid-2021, yet hesitancy persisted due to safety concerns and mistrust in science [26]. For instance, studies by Smith [27] and Johnson et al. [28] have highlighted the role of misinformation and concerns about vaccine safety in shaping vaccine-related discourse. These recurring themes underscore the importance of addressing specific concerns and providing accurate information to mitigate vaccine hesitancy effectively [6]. Moreover, the varying confidence levels for the different brands among Filipinos led to concerns over the ingredients of vaccines [29].

This study revealed an interesting pattern regarding the sources of information cited in vaccine-hesitant comments. We observed that vaccine-hesitant individuals relied on a broader array of informational sources compared to those expressing support for vaccination. Among the frequently cited sources were “YouTube users”, “politicians”, “clinicians”, and “scientific papers”. This finding aligns with prior research highlighting the diverse range of information channels influencing vaccine attitudes and beliefs. Betsch et al. [18] have discussed the role of social media platforms and political discourse in shaping vaccine-related opinions. These results emphasize the importance of engaging with various stakeholders, including healthcare professionals and policymakers, to address vaccine hesitancy effectively [6,30].

Within the realm of vaccine-hesitant comments, our analysis identified distinct discourse types that shed light on the various perspectives and concerns expressed. These discourse types included “negationists”, who outright denied the efficacy or safety of vaccines, “institutional” critics who expressed skepticism towards health authorities and vaccination programs, “preventive” individuals who emphasized alternative health practices, “politics” focused on political ideologies and conspiracy theories, “pharmaceutic” critics who questioned the motives of pharmaceutical companies, and “questioning” individuals who sought information and clarification. This classification aligns with previous research exploring the multifaceted nature of vaccine hesitancy [6,31,32], highlighting the diverse range of concerns and beliefs underlying vaccine-hesitant attitudes. Finally, our analysis also found that mentions of government representatives were the most frequently observed within the comments, indicating the perceived influence and role of governmental entities in shaping vaccine-related discourse. Previous research has shown how political ideology is a predictive factor for perception of vaccines [33].

While this study contributes to understanding vaccine hesitancy discourse on social media platforms, it is important to acknowledge certain limitations. Firstly, the analysis focused specifically on YouTube comments may not fully represent the broader population’s attitudes towards COVID-19 vaccination. The small sample size of videos analyzed may capture just a fraction of the broader public discourse surrounding the COVID-19 vaccination campaign in the Philippines. This study’s focus on the Philippines limits generalizability to other countries or regions with different socio-cultural contexts. Moreover, the analysis relied on publicly available comments, which may not reflect the views of individuals who chose not to engage in online discussions. Furthermore, this study primarily examined engagement levels and discourse themes, without exploring the underlying motivations and beliefs that drive vaccine hesitancy. Another limitation of this study is the unique context of the COVID-19 pandemic, which introduced unprecedented emotional, social, and ethical dimensions to vaccination discourse due to its global scale, rapid vaccine development timeline, and implementation of emergency use authorizations. These factors created a distinct public communication environment characterized by heightened anxiety, uncertainty, and politicization not typically present in other vaccination contexts. Furthermore, healthcare workers (HCWs) occupied a unique position in the public debate—simultaneously serving as both authority figures and potential vaccine recipients whose own hesitancy could influence broader public perception. While research from Europe indicates that HCW confidence significantly impacts public trust in vaccines [34], our study did not specifically analyze the role of healthcare professionals in the Philippines’ vaccination discourse. This represents an important avenue for future research, particularly given the heightened social media engagement observed during pandemic conditions. These contextual factors limit direct extrapolation of our findings to non-pandemic vaccination campaigns. Future research could consider incorporating qualitative methods such as interviews or surveys to gain a deeper understanding of the reasons behind vaccine hesitancy. Longitudinal studies could also be conducted to track changes in vaccine-related discourse over time. Finally, considering the dynamic nature of social media platforms, future research should also explore the effectiveness of interventions aimed at countering vaccine hesitancy and misinformation within these digital spaces.

In conclusion, this study provides valuable insights into vaccine hesitancy discourse on YouTube and its impact on public engagement during the COVID-19 vaccination campaign in the Philippines. The findings highlight the prevalence of negative attitudes towards vaccination and the influential role of social media platforms in shaping public perceptions. The identified discourse types, themes, and information sources shed light on the complexity of vaccine hesitancy and emphasize the need for targeted communication strategies that address specific concerns and provide accurate information. Future research should further explore the underlying motivations behind vaccine hesitancy and evaluate the effectiveness of interventions in combating misinformation on social media platforms.

## Figures and Tables

**Table 1:** Variables used in the comments coding.

Sources of information	Scientist—comments mentioning sources from an expert/scholar Politician—comments mentioning sources of information from a government official/staff Clinician—comments mentioning sources of information from a doctor/clinician Scientific Paper—comments mentioning sources of information from a journal article/book Websites—comments mentioning sources of information from a website Pharma/Company—comments mentioning sources of information from a pharmaceutical company Users—comments based on his/her observations/experiences Others—other sources of information not listed above
Tone towards vaccination	Positive—if the statement clearly recommended vaccination Negative—if arguments are put forward against vaccination Neutral—if there are not statements either for or against vaccination Not applicable
Themes of messages comments	Ingredients of vaccines—comments about elements of vaccines or issues concerning vaccine ingredients; Ex: The vaccine probably contains mercury and other chemicals including raw eggs. Adverse effects—comments expressing possible side effects of a vaccine; Ex: You will feel itchiness or swelling at the injection site. Pharma industry—comments expressing perceived influence of pharma industry rushing vaccine production; Ex: The Pharma industry is getting so much profit. Economic interests—comments expressing concerns on economic gain/interests; Ex: Why would I get the COVID-19 vaccine? It will just make big companies get rich from it. Social population control—comments expressing COVID-19/vaccination as a way of population control; Ex: COVID-19 is just created to lessen the population of the world. Religion—expressing religious beliefs and role of religion; Ex: This is what the bible states as the end of the world where people suffer. Health authorities—comments mentioning the health authorities; Ex: Vaccination is being organized by the national healthcare services. Disease prevalence—comments expressing COVID-19 vaccine as unnecessary because the virus is not dangerous; Ex: It’s already hit my house, both my wife and I are at very high risk, no hospital for either of us and yet here we are!) School—comments related to schools and how they are hosting and managing COVID-19 infection; Ex: We don’t want our children to go back to school. Conspiracies—comments that either support or oppose conspiracy narratives; Ex: It was a product of an experiment from a lab in China. Development speed—comments that either support or oppose the how the vaccine was developed; Ex. COVID-19 vaccine were fast-tracked to benefit pharma companies.
Vaccine-hesitant comments	Vaccine refusers—comments stating that they would not take a COVID-19 vaccine; Vaccine hesitators—expressing reservations and doubts about vaccination, or delaying taking the COVID-19 vaccine; Other—not expressing an opinion on vaccine hesitancy
Public discourse classification	Prevention—comments expressing intention to get the COVID-19 vaccine so that they can prevent the disease and be able to return to their normal life; Ex: Taking the vaccine will prevent more cases and we can go back to normal. Politics—stating around the perceived influence of political pressure rushing vaccine production or the political management of the vaccination programs; Ex: Why would I get the Chinese vaccine? Let the President puppet of China get it first. Negationist—expressing denial or invalidating the effectiveness of the COVID-19 vaccine Fear—if the comment express worries of danger or harm from taking the COVID-19 vaccine Institutional—comment expressing support or trust to institutions such as health institutions Contradiction (nonsense)—comments expressing opposing views about the COVID-19 vaccine Humor/Ironic—comments expressing the COVID-19 vaccine as unnecessary because the virus was not dangerous Gratitude—if the comment express appreciation for and a desire to return kindness Pharmaceutic—comments expressing trust/mistrust in the pharmaceutical industry behind the vaccine Economic—comments expressing perceived influence of economic gains in taking the COVID-19 vaccine Questioning—comment expressing so as to elicit more information about the vaccine Scientific—comments expressing trust in the science behind the vaccines; Ex: If the doctors approve a vaccine, then it’s good. Motivational—comments promoting the desire or willingness to take the vaccine Volunteering—comments expressing openness to take the vaccine without being paid Trust—comments expressing trust in the vaccine and a real intent to get the COVID-19 vaccine to protect others and return to normal Protest—comments expressing disapproval or objection to the statements or facts about the COVID-19 vaccine Hope—comments expressing anticipation and confidence about the efficacy of the COVID-19 vaccine

**Table 2:** Engagement level and vaccine hesitancy.

		Vaccine Hesitancy		Total
		Yes	No	
Engagement level	0	450	128	578
	1–2	60	11	71
	3–4	37	3	40
	5–10	28	3	31
	11–94	18	3	21
Total		593	148	741

Mann–Whitney U test = 39,103; *p* < 0.05.

**Table 3:** Themes among the vaccine-hesitant comments.

		N (%)
Themes	Ingredients of vaccines	182 (25)
	Health dept. control	156 (21)
	Pharma interests	144 (20)
	Adverse effects	128 (18)
	Low risk	33 (5)
	Economic interest	32 (4)
	Religion	22 (3)
	Conspiracies	14 (2)
	Development speed	10 (1)
	Population control	9 (1)
Total		730

**Table 4:** Source categories.

		Hesitant N (%)
Sources	Scientist	5 (4)
	Politician	25 (19)
	Clinician	13 (10)
	Scientific paper	10 (8)
	Websites	5 (4)
	Pharma/company	1 (1)
	Users	41 (31)
	Others	32 (24)
Total		132 (100)

**Table 5:** Discourse type.

		Hesitant N (%)
Discourse	Negationist	136 (16)
	Institutional	116 (14)
	Preventive	82 (10)
	Politics	81 (9)
	Questioning	61 (7)
	Pharmaceutic	57 (7)
	Contradiction	53 (6)
	Fear	50 (6)
	Humor/Ironic	45 (5)
	Scientific	41 (5)
	Gratitude	39 (5)
	Protest	32 (4)
	Economic	20 (2)
	Motivational	16 (2)
	Truth	13 (2)
	Volunteering	11 (1)
	Hope	2 (0.5)
Total		855

**Table 6:** Mentions made in the comments.

Category	Name	N
Government	Duque President Duterte Vice president, Galvez Department of Health, Politician, Senators, VP Robredo, Indonesian President Congress, Roque, Govt. Officials, President of Turkey, Malacanang, Marcos, Leni, Sinas	28 18 17 4 2 1
Anonymous	Military police Aileen Siaboc, angel of death, Bishop priest, Lorenzana, Kun Chi, Levy, Susan Militante	2 1
Country	China EU, US	4 1
Healthcare services	Pharma industry, Director of PGH, Clinician	1
Religious group	Religious group	1
	Total	108

## Data Availability

The data will be made available in the article.
